# Use of the Multivariate Discriminant Analysis for Genome-Wide Association Studies in Cattle

**DOI:** 10.3390/ani10081300

**Published:** 2020-07-29

**Authors:** Elisabetta Manca, Alberto Cesarani, Giustino Gaspa, Silvia Sorbolini, Nicolò P.P. Macciotta, Corrado Dimauro

**Affiliations:** 1 Dipartimento di Agraria, Università degli Studi di Sassari, 07100 Sassari, Italy; elimanca@uniss.it (E.M.); acesarani@uniss.it (A.C.); ssorbolini@uniss.it (S.S.); macciott@uniss.it (N.P.P.M.); 2 Dipartimento di Scienze Agrarie, Forestali e Ambientali, Università degli studi di Torino, 10095 Grugliasco, Italy; giustino.gaspa@unito.it

**Keywords:** multivariate statistics, association study, carcass trait, meet quality trait

## Abstract

**Simple Summary:**

In the traditional single marker regression approach for genome-wide association studies, if the number of involved individuals is small and the number of single nucleotide polymorphisms (SNPs) to be tested is very large, the probability of getting a significant association simply due to chance becomes enormous. Other techniques, such as the Bayesian methods, require several a priori assumptions, as an a priori posterior inclusion probability threshold, that can limit their effectiveness. In the present study, a multivariate algorithm able to partially overcome this problem was proposed. On simulated data, with 3000 individuals, only 13 and 3 quantitative trait loci (QTLs) were obtained with the single marker regression and the Bayesian approaches, respectively. On the other hand, the multivariate algorithm detected 65 QTLs in the same scenario. The gap between the single marker regression and the multivariate methods slowly decreased as the number of animals increased. This figure was also confirmed on real data.

**Abstract:**

Genome-wide association studies (GWAS) are traditionally carried out by using the single marker regression model that, if a small number of individuals is involved, often lead to very few associations. The Bayesian methods, such as BayesR, have obtained encouraging results when they are applied to the GWAS. However, these approaches, require that an a priori posterior inclusion probability threshold be fixed, thus arbitrarily affecting the obtained associations. To partially overcome these problems, a multivariate statistical algorithm was proposed. The basic idea was that animals with different phenotypic values of a specific trait share different allelic combinations for genes involved in its determinism. Three multivariate techniques were used to highlight the differences between the individuals assembled in high and low phenotype groups: the canonical discriminant analysis, the discriminant analysis and the stepwise discriminant analysis. The multivariate method was tested both on simulated and on real data. The results from the simulation study highlighted that the multivariate GWAS detected a greater number of true associated single nucleotide polymorphisms (SNPs) and Quantitative trait loci (QTLs) than the single marker model and the Bayesian approach. For example, with 3000 animals, the traditional GWAS highlighted only 29 significantly associated markers and 13 QTLs, whereas the multivariate method found 127 associated SNPs and 65 QTLs. The gap between the two approaches slowly decreased as the number of animals increased. The Bayesian method gave worse results than the other two. On average, with the real data, the multivariate GWAS found 108 associated markers for each trait under study and among them, around 63% SNPs were also found in the single marker approach. Among the top 118 associated markers, 76 SNPs harbored putative candidate genes.

## 1. Introduction

Genome-wide association studies (GWAS) are mainly aimed at understanding the genetic background of complex traits by relating a large number of single nucleotide polymorphism (SNP) genotypes to observed phenotypes. Traditionally, GWAS is carried out by using the single marker regression model that includes both fixed and random effects. We will refer to this approach as traditional GWAS (T-GWAS). When the number of SNPs to be tested is very large, the probability of getting a significant association simply due to chance becomes enormous. This multiple effect is often controlled by using the Bonferroni’s correction that, however, requires that all tests are independent of each other [1]. In genomic data analysis, this statement generally does not hold because as the marker density increases, tests become more correlated due to the linkage disequilibrium among adjacent SNPs. Common alternatives to Bonferroni’s correction are the permutation method or the false discovery rate (FDR) procedure [2,3]. The former method essentially corrects for the number of expected false discoveries. However, it can be too conservative, depending on the fraction of discoveries that are tolerated to be false.

Evidently, a T-GWAS has a high probability of obtaining reliable results if the number of involved individuals is large. Frequently, this number is small due to the high genotyping costs or to difficulties in phenotype recording (methane emission or residual feed intake, for example). Moreover, some species or breeds, as local breeds, have an intrinsically small population size, thus conditioning a priori any association study. The combination of the great number of markers and the small number of genotyped individuals leads to obtain very few associations.

A significant SNP usually explains only a small fraction of the genetic variance of quantitative traits [4,5]. Genetic differences generally are not localized in a single locus but often involve the surrounding part of the genome too. The analysis of the correlation structure between SNPs may offer useful insights for finding chromosomal segments associated to the phenotypic expression of traits of interest. One example, that have obtained encouraging results, is the Bayesian approach (B-GWAS in the present research) that, originally proposed for genomic predictions [6], has been used for GWAS as well [7,8]. However, these approaches require several a priori assumptions that, sometimes, can limit their use. An alternative to T-GWAS and B-GWAS could be a multivariate approach that, by definition, is able to simultaneously analyze the correlation structure of multiple variables [9], i.e., the marker-genotypes in this study.

The aim of the present research was to propose a new statistical approach to perform a GWAS, able to overcome most of difficulties encountered with T and B-GWAS, especially when few genotyped animals are involved. The method, called multivariate GWAS (M-GWAS), exploits three multivariate discriminant techniques that are successively applied to the data: the canonical discriminant analysis (CDA), the discriminant analysis (DA) and the stepwise discriminant analysis (SDA). The above listed techniques are not considerably affected by the number of involved individuals, do not require any statistical test (with a *p*-value) to declare a SNP associated to a particular trait and are able to analyze hundreds of thousands of SNPs simultaneously.

These statistical procedures have already been used in genomics to detect the pools of markers useful to trace cattle and sheep breeds [10,11], to explore the relationships between milk characteristics [12], to highlight the signatures of selection [13], and to detect the carriers of recessive haplotypes [14].

The rationale of M-GWAS is based on the idea that individuals with different phenotypic values of a specific trait share different allelic combinations for genes involved in its determinism. Genetic differences between animals belonging to different groups (i.e., with low or high phenotype values) are highlighted by using the three multivariate discriminant procedures listed above.

The proposed M-GWAS algorithm was tested both on a simulated cattle population and on the real data from a beef cattle breed previously analyzed with a T-GWAS, whose results were published by Sorbolini et al. [15].

## 2. Materials and Methods

### 2.1. The Data

A cattle population was created using QMSim [16]. Data were generated to mimic a beef trait with phenotypic variance of 1.0 and heritability of 0.5. The simulated genome consisted of 29 autosomes with 56,996 evenly allocated biallelic SNPs and 203 randomly distributed biallelic QTLs whose heritability was fixed at 0.3. The remaining quota of heritability was due to a simulated polygenic effect. The recurrent mutation rate of SNPs and QTLs were assumed to be 2.5 × 10^−5^ per locus per generation [17]. The historical population was obtained with the creation of 1000 generations. Ten recent generations were simulated starting from 100 males and 4000 females, i.e., one male for 40 females, with the last 3 generations being genotyped. Other parameters of the simulated population were: sire and dame replacement rate fixed at 0.2 and 0.3, respectively; positive assortative with randomly selected animals was adopted as mating design; 0.5 proportion of male progeny. The final dataset contained a simulated bovine population with 12,000 animals, 6043 males and 5957 females. From this data, 20 sub-datasets, each containing an increasing number of animals (Table 1), were randomly extracted to compare the ability of M-GWAS as regards T-GWAS and B-GWAS in detecting associated SNPs across different sample sizes.

The real data used in the present research was previously analyzed by Sorbolini et al. [15] who carried out a T-GWAS on 409 young Marchigiana bulls belonging to 117 commercial herds. Animals were genotyped by using the Illumina 50 K BeadChip assay. The aim of that study was the detection of markers significantly associated with carcass and meat traits. Animals were slaughtered at an age ranging from 16 to 24 months. Over 10 traits, 7 of them highlighted markers significantly associated in the T-GWAS: body weight (BW), average daily gain (ADG), carcass weight (CW), dressing percentage (DP), shank circumference (SC), head weight (HW) and pH at slaughter (pH). No association was found for the remaining three traits (carcass conformation (CC), pH at 24 h after slaughter (pH24h) and skin weight (SW)). In the present study, the first seven traits (BW, ADG, CW, DP, SC, HW, and pH) were used to test the M-GWAS approach on the real data. Then, the multivariate method was applied to the remaining three traits (CC, pH24h and SW) to ascertain if this approach was able to detect associations.

### 2.2. The Traditional and the Bayesian GWAS Approaches

Phenotypes analyzed in this study were pre-adjusted both for fixed and random effects. For the simulated phenotype, the following mixed linear model was used:

(1) Y=G+S+a+e

where Y = the simulated phenotype; G = fixed effect of generation (10 levels); S = fixed effect of sex (2 levels); a = random additive genetic effect of the animal; e = random residuals. Residuals were used as the corrected phenotype in the subsequent GWAS analyses.

The model used for the real data was [15]:

(2) Y=D+bAGE+a+h+e

where Y = the considered phenotype (10 traits, one trait at a time); D = fixed effect of slaughter date (46 levels); bAGE = fixed covariable of age at slaughter in month; a = random effect of animal; h = random effect of herd (117); e = random residuals. As before, the residuals from the model applied to the 10 traits were used as corrected phenotypes in the GWAS.

Animals both in the 20 simulated datasets and in the real data were ranked according to their corrected phenotypes and divided into low phenotype (LP) and high phenotype (HP). The two groups contained an equal number of individuals.

For the simulated data, the T-GWAS was developed using PLINK software according to Marees et al. [18]. Since the animals came from the same population, multidimensional scaling was performed by extracting from SNP-variables the first 10 principal components used as effect in the GWAS in order to correct for the relationship among animals. In this way, false positive associations due to family effect were constrained. *p*-values of each SNP were corrected using the FDR procedure [2,3].

Among the various Bayesian methods commonly used to identify marker-traits associations, the BayesR approach was preferred to the others for its ability in simultaneously accommodating SNPs with large, small, and no effect [8]. Simulated data were analyzed using the BayesR software (https://cnsgenomics.com/content/software). Briefly, in the Bayesian R approach, the SNP effects are drown from one of four normal distributions with N (0, 0 σ^2^_g_), N (0, 0.0001 σ^2^_g_), N (0, 0.001 σ^2^_g_) and N(0, 0.01 σ^2^_g_), where σ^2^_g_ is the additive genetic variance. The procedure classifies SNPs, according to their posterior inclusion probability, into one of the four distributions. Two different strategies were adopted to the select associated markers. In the first, one SNP was declared associated to the trait under study if its posterior inclusion probability threshold was greater than 0.30 [19]. In the second, called B-GWAS2, the markers were ranked according to their descending posterior inclusion probability value and, for each dataset, the first n-SNPs, with n equal to the number of associated markers obtained in the corresponding M-GWAS, were retained and considered associated. So, for example, if in the sample with 2000 animals the M-GWAS found 100 associated SNPs, then when the B-GWAS2 was applied to the same dataset, the first 100 markers, that have the greater posterior inclusion probability value, were declared associated.

For real data, the T-GWAS results were those reported by Sorbolini et al. [15].

### 2.3. The Multivariate Statistical Analysis

Three multivariate discriminant techniques were successively applied to the data: the canonical discriminant analysis (CDA), the discriminant analysis (DA) and the stepwise discriminant analysis (SDA). CDA is a multivariate dimension-reduction technique whose main objective is the determination of relationships among a categorical variable and a list of independent variables. In particular, CDA tests whether the independent variables are able to identify the groups listed in the categorical variable. In our research, the categories were HP and LP, whereas the independent variables were the SNP genotypes. CDA derives a set of new variables, called canonical functions (CAN) that are linear combinations of the original characters. The structure of the generic CAN is:

(3) $$CAN=d_{1}X_{1}+d_{2}X_{2}+.....+d_{n}X_{n}$$

where, X_i_ are the original variables (SNP genotypes in the present study), and d_i_ are the canonical coefficients (CNC), i.e., the loadings of original variables in composing the CAN. The greater a CNC (in absolute value), the larger the contribution of the corresponding variable to the CAN.

In general, if k-groups are involved, the CDA extracts k-1 CANs. In this research, having two groups (HP and LP), only one CAN for each phenotype was obtained. The separation between the groups can be assessed by the means of the Mahalanobis distance and the corresponding Hotelling’s T-squared test [20]. This test, however, can be developed only if the (co)variance matrix of the data is nonsingular. In most genomic studies, the number of involved animals (rows of data matrix) is markedly lower than the number of SNPs (columns), even for each single chromosome. In these conditions, the (co)variance matrix does not have a full rank [21]. A reduction of the variable space is, therefore, required. This objective can be achieved following the suggestions of Dimauro et al. [10]. In each chromosome, CNCs are ranked according to their absolute value and the first m-SNPs are retained. Selected markers are then joined and used to develop a new CDA run that, in this step, is a genome-wide CDA. The optimum space dimension of the independent variables is finally obtained by using the SDA, i.e., a statistical technique specifically conceived to select the subset of variables that better separate groups. The procedure gives as output the maximum number of linearly independent markers, for each phenotype. Hence, when the genome-wide CDA is developed, both the Mahalanobis’ distance and Hotelling’s test can be evaluated.

The DA was used to classify animals into groups (LP or HP in this research). In DA, the CAN (extracted in the CDA) is applied to each individual, thus producing a discriminant score. An animal is assigned to a particular group if its discriminant score is lower than the cutoff value obtained by calculating the weighted mean distance among the group centroids [22].

### 2.4. The Multivariate Algorithm for SNP Association

For each phenotype (both real and simulated), after the LP and HP groups were obtained, the M-GWAS method can be summarized as follows:

1. The CDA was applied by chromosome and the mean and the standard deviation of the absolute value of the CNCs in the CAN were calculated. Only markers whose CNC’s absolute value is greater than the mean plus one standard deviation are retained in each chromosome;
2. Markers selected in the 29 autosomes were joined and the SDA was applied to obtain the maximum number of linearly independent markers. The resulting SNPs are, in consequence, not in complete linkage disequilibrium;
3. Markers selected in the previous step were first used, in a new run of CDA, to test the effective separation of LP from HP, and then in a run of DA to ascertain if animals were correctly assigned to the group of origin (HP and LP);
4. Finally, the minimum number of markers able to significantly discriminate LP from HP and, in the same time, correctly assign animals to the true group of origin was selected. This objective is achieved applying, in sequence, the CDA and the DA and deleting, in a recursive procedure, SNPs with the lower CNC absolute values. The procedure stopped when Hotelling’s test was still highly significant (*p*-value < 0.001) and at the same time, all the animals were correctly assigned to LP and HP. The obtained SNPs were considered associated to the phenotype.

Statistical analyses in the M-GWAS were developed by using the MIXED, CANDISC, DISCRIM and STEPDISC procedures of SAS, release 9.4 (SAS Inst. Inc., Cary, NC, USA). The SAS code and data to develop the M-GWAS are available in Supplementary Materials.

For the simulated data, the ability of the GWAS approaches in detecting associations was assessed comparing the obtained number of the true associated SNPs, i.e., the markers close to one of the 203 (known) simulated QTLs (250 Kb upstream and downstream of the QTL position). Since a QTL could be associated with more than one SNP, the effective number of detected QTLs was considered the most important index for comparing the GWAS methods.

### 2.5. Annotation and Gene Discovery Analysis

With real data, being QTLs and their position across the genome, unknown, the ability of M-GWAS in detecting SNPs strongly associated to some putative gene was tested through a gene discovery study. With this aim, for each of the 10 traits under study, a restricted pool of highly associated SNPs was selected. These markers were obtained by ranking the associated SNPs and fixing an arbitrary CNC threshold such as the number of remaining markers, which was, at the end, around 12 for each trait. Annotated genes were identified from the UCSC Genome Browser Gateway (http://genome.ucsc.edu./) and National Centre for Biotechnology Information (NCBI) (www.ncbi.nlm.nih.gov) databases. Intervals of 250 Kb upstream and downstream of each SNP were considered. Gene-specific functional analyses were performed by GeneCards (www.genecards.org) and NCBI databases consultation. The biological function of each annotated gene (and the related proteins) contained in the significant genomic regions was studied by means of an accurate literature search. Gene names and symbols were derived from the HUGO Gene nomenclature database (www.genenames.org).

## 3. Results

### 3.1. Simulated Data

Results of M-GWAS and T-GWAS applied to 20 sub-datasets extracted from the simulated population are summarized in Table 1, that reports for each sample the number of associated SNPs, the true associated SNPs and the detected QTLs, respectively. A similar table (Table 2) was also added to show the results obtained in B-GWAS1 and B-GWAS2. For instance, the associated markers were the minimum number of SNPs able to significantly separate LP from HP and to correctly assign the animals to the true group of origin. True associated SNPs were those that, in the nearby, harbored a QTL.

**Figure 1:**
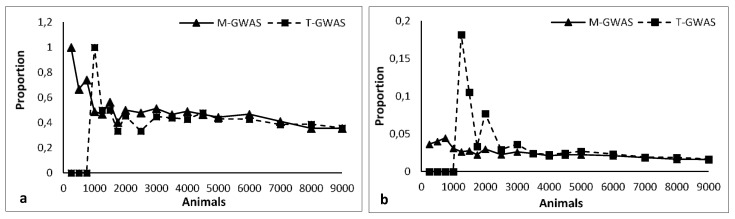
The proportion of detected QTLs with respect to the true associated (**a**) and false associated (**b**) markers for both multivariate (M-GWAS) and traditional (T-GWAS) GWAS as the number of involved animals increases.

Excluding the last two datasets (8000 and 9000 animals), M-GWAS always detected a greater number of QTLs than the T-GWAS did. When the animal sample size was small, from 250 to 750, T-GWAS was not able to highlight any association. Only 1 QTL was found with 1000 individuals. In any case, M-GWAS detected a larger number of QTLs than T-GWAS when the involved animals ranged from 250 to 3000. The gap in the QTL detection between M-GWAS and T-GWAS slowly decreased as the number of animals increased. When 8000 individuals were used, this difference was only of two QTLs. The proportion of detected QTLs with respect to the true associated markers (Figure 1a) was almost always greater in M-GWAS than in T-GWAS. However, as the number of involved animals increased, this proportion tended to converge. A completely opposite trend occurred when the proportion of detected QTLs with respect to false associated markers (Figure 1b) was considered.

Results from B-GWAS2 (Table 2) were quite like those obtained by M-GWAS and sometimes better, whereas B-GWAS1 was the method that gave the worst results. In this case, the number of detected QTLs was very small, ranging from zero with a sample size of 250 animals, to seven with 9000 individuals.

Table 3 displays the number of QTLs that were simultaneously detected by the M-GWAS and other approaches. In the scenario M-GWAS vs. T-GWAS, the first common QTL was found with a sample size of 1000. However, as the number of individuals grew, the number of common QTLs increased. With 9000 animals, 78 QTLs were simultaneously detected by the two techniques. M-GWAS found very few common QTLs with B-GWAS1, whereas with B-GWAS2, ever since 500 animals, four common QTLs were detected. This number increases until 90 common QTLs in the dataset with 9000 animals.

### 3.2. Real Data

**Figure 2:**
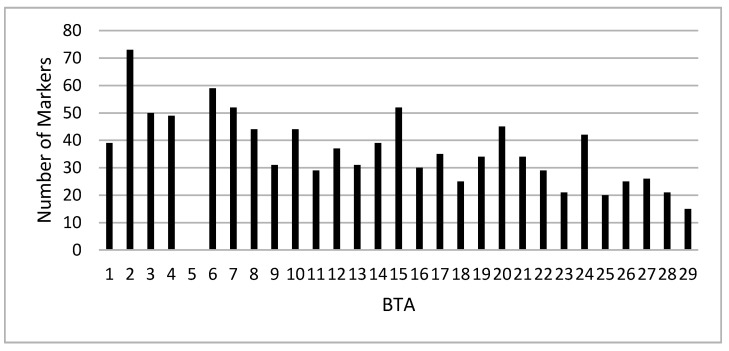
Distribution across the genome of 1031 markers selected in the multivariate GWAS for the first seven traits (body weight (BW), average daily gain (ADG), carcass weight (CW), dressing percentage (DP), shank circumference (SC), head weight (HW) and pH at slaughter (pH)).

Results found by Sorbolini et al. [15] with the T-GWAS were compared to those obtained with the multivariate approach. The M-GWAS selected, on the whole for the first seven traits, 1031 markers spanning the entire genome (Figure 2). The largest number of SNPs (73) was found on Bos taurus autosome BTA2 followed by BTA6 (59). The lowest number (15) was located on BTA29. No significant marker was observed on BTA 5.

On average, around 190 linearly independent SNPs (Table 4) for each phenotype were retained using the M-GWAS. The subsequent genome-wide CDA, developed for each trait, significantly separated the HP from the LP (Hotelling’s *T*-test *p*-value < 0.001) and the DA correctly assigned all the animals to the correct group.

Considering the markers associated in the T-GWAS (Table 3), around 63% of them were confirmed by the multivariate approach. For example, most of T-GWAS SNPs were identified by M-GWAS for BW, DP and ADG. Only one over five markers for pH, and six over 13 SNPs for SC were obtained by M-GWAS.

The minimum number of SNPs able to significantly discriminate LP from HP and correctly assign animals to groups is also reported in Table 3. These markers represent, for each phenotype, the most discriminant SNPs and their number ranges from 139 for ADG to 94 for BW.

The M-GWAS was then applied to the three traits (CC, SW and pH24h) that in the T-GWAS did not have any associated SNP. The procedure selected 141, 148 and 176 linearly independent markers for CC, SW and pH24h, respectively, whereas the minimum number of SNPs able to significantly discriminate groups was slightly lower (122, 129 and 142 for CC, SW and pH24h, respectively).

**Figure 3:**
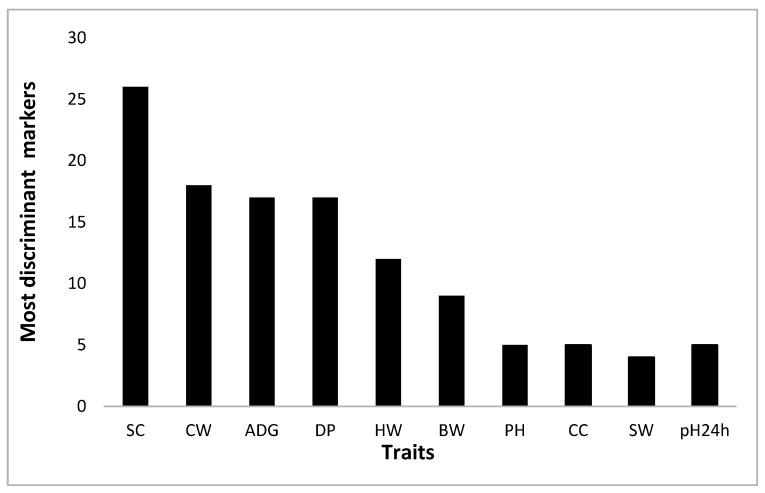
Number of most discriminant markers selected by the multivariate GWAS approach for the 10 traits under study. BW = body weight, ADG = average daily gain, CW = carcass weight, DP = dressing percentage, SC = shank circumference, HW = head weight, pH = pH at slaughter, CC = carcass conformation, SW = skin weight, pH24h= pH 24 h after slaughter.

Finally, a restricted group of top discriminant SNPs (118) was selected by using a CNC arbitrary threshold equal to 0.25 (Figure 3). Only these markers were submitted to gene discovery.

### 3.3. Gene Discovery

Among the top discriminant markers, only 76 over 118 SNPs harbored genes in their boundaries (Table S1).

Twenty-six significant highly discriminant markers were found to be associated with SC. For example, in BTA 2, close to ARS-BFGL-NGS-71755 and to BTB-02054371, the gene Titin (TTN) that encodes a large protein of striated muscle and the protein activator of interferon-induced protein kinase (EIF2AK2) can be found, respectively.

Eighteen highly discriminant SNPs were found to be associated with CW but only one of them (UA-IFASA-6018 on BTA22) was close to two interesting genes: the transketolase (TKT) and protein kinase C delta (PRKCD).

For ADG, seventeen highly discriminant SNPs, distributed across ten chromosomes, were selected. For example, on BTA 3, four candidate genes close to marker ARS-BFGL-NGS-119955 were considered as interesting: PAS domain-containing serine/threonine kinase (PASK), the high density lipoprotein-binding protein (HDLBP), the inhibitor of growth family member (ING5) and the deoxythymidylate kinase (DTYMK). On BTA10 instead, two putative genes were found close to marker ARS-BFGL-NGS-116295: lactase-like (LCT) and a member of small nuclear RNA-activating complex polypeptide 5 (SNAPC5).

Seventeen highly discriminant SNPs were found associated with DP. In particular, on BTAs 18 and 22 two loci were identified as candidate genes, the syntrophin beta 2 (SNTB2) and the solute carrier family 6 member 6 (SLC6A6), respectively.

In addition, for HW, several (12) highly discriminant markers were found. Among the others, on BTA 7 at 44.8 Mb three putative candidate genes involved in the brain biology were annotated, the Basigin (OK blood group) (BSG), the hyperpolarization-activated cyclin nucleotide gated potassium channel 2 (HCN2) and the follistatin-like 3 (FSTL3).

A total of nine highly discriminant SNPs distributed across six autosomes were found associated with BW. On BTA 4, the annotated sequence nearest to the marker BTB-00182742 was the phosphoinositide 3-kinase gamma (PIK3GC). On BTA 22 the marker Hapmap41774-BTA-121358 was already reported as significant for CW.

Only five highly discriminant markers were found to be associated with pH. For exanple, on BTA18, the phosphorylase kinase regulatory subunit beta (PHKB) was associated with the marker ARS-BFGL-NGS-24006, whereas on BTA 23 at 9.1 Mb, close to the marker Hapmap38418-BTA-146026, the gene peroxisome proliferator-activated receptor delta (PPARD) was detected.

Moreover, for CC, five highly discriminant associated markers were found. In their neighbor fourteen genes were detected. Among them, for example, on BTA 1 the TBC1 domain family member 5 (TBC1D5) gene was retrieved.

For SW, four highly discriminant-associated markers were found with a total of 15 genes in the nearest of them. Among these genes, two, the BAF chromatin remodeling complex subunit BCL11A (BCL11A) and PTEN-induced kinase 1 (PINK1) were mapped on BTA11 and BTA2.

Finally, for pH24h, five highly discriminant SNPs were found with 31 genes in their boundaries. The chromosome with the highest number of genes was BTA29 (11), followed by BTA19 (10). Other genes were found on BTA9 and 10 (three genes each one) and BTA27 (four genes). Most of these genes were reported in literature to be associated with meat traits.

## 4. Discussion

### 4.1. Simulation Study

The M-GWAS approach was tested and compared with T-GWAS and B-GWAS using genotypes from a simulated bovine population of 12,000 individuals. All the statistical techniques were applied to 20 random sub-samples extracted from the population with an increasing sample size (Table 1 and Table 2). In M-GWAS, for each dataset, the markers selected in the 29 autosomes were used in a run of the SDA to obtain the maximum number of genome-wide linearly independent SNP-variables. This number is always lower than the number of involved individuals [21]. The CDA developed using the selected markers was able to significantly separate the LP from the HP and the DA correctly assigned all the animals to the two groups. Despite B-GWAS1, where the associated SNPs are obtained fixing an arbitrary posterior inclusion probability cutoff [19], in M-GWAS, no a priori threshold was imposed. Associated SNPs were selected with a recursive procedure that among linearly independent markers, retained the minimum number of SNPs able to significantly separate groups and in the same time, correctly assign animals to LP or HP. In simulated data, the number of QTLs (203) and their position across the genome was known. Therefore, among their associated markers, SNPs that in the nearby harbored a QTL were flagged as true associated (Table 1 and Table 2). On the other hand, more than one marker can be associated to the same QTL, and of course, the number of true associated markers could be, generally, greater than the number of detected QTLs.

Among the four statistical approaches, the B-GWAS1 gave the poorest results, with a maximum number of detected QTLs equal to seven with 9000 animals. If these data were compared with that obtained by B-GWAS2, it appears obvious that the reason of the great difference between the number of detected QTLs by the two methods was due to the a priori posterior inclusion probability threshold that was imposed on B-GWAS1 [19]. The need of an arbitrary threshold to declare associations often limits the use of Bayesian methods in GWAS [23]. In B-GWAS2, the markers were ranked according to their descending posterior inclusion probability value and for each dataset, an equal number of associated markers obtained by M-GWAS were retained. In this condition, B-GWAS2 detected a greater number of QTLs than M-GWAS for almost all the sample sizes (Table 1 and Table 2).

Table 1 displays the results both for M-GWAS and T-GWAS. The number of detected QTLs was greater in M-GWAS than in T-GWAS. When the number of involved animals was low (<750) T-GWAS did not highlight any associated maker, whereas M-GWAS detected both true associated SNPs and QTLs (Table 1) in a sample with 250 individuals. Moreover, M-GWAS found a number of true associated SNPs and QTLs largely greater than T-GWAS in the scenarios from 250 to 3500 animals. In larger sub-datasets, these numbers tend to converge in the two approaches, and with 9000 individuals they were nearly equal.

Apart from the initial scenarios where M-GWAS clearly prevailed on T-GWAS, the proportion of detected QTLs with respect to true associated markers (Figure 1a) was nearly equal for the two techniques, with a slight preponderance of M-GWAS. This figure was very similar when the proportion of detected QTLs and falsely associated SNPs was considered. In this case, T-GWAS slightly prevailed on M-GWAS. The two techniques showed nearly equal performances when the number of involved animals was great (more than 8000).

With a reduced number of animals, the proportion of false positive markers was greater for M-GWAS than for T-GWAS. These values slowly met as the number of involved individuals increased. However, considering that a lot of false associations can be discarded when the gene discovery is developed, the high number of false positive SNPs does not invalidate the multivariate procedure. In any case, the overall results indicate that especially when the number of genotyped animals is low, the M-GWAS could be preferred to T-GWAS.

Several QTLs were simultaneously detected by M-GWAS and the other methods (Table 3). Results suggested that different methods detected, at least in part, QTLs in different regions of the genome. For example, comparing M-GWAS and T-GWAS in the scenario with 5000 animals, 42 QTLs were commonly detected, whereas 54 and 31 QTLs were exclusively found by M-GWAS and T-GWAS, respectively. As a consequence, in this scenario, if the two methods were used, 127 over 203 QTLs could be detected. It follows that the combined use of the two approaches in the same research, especially when the number of involved animals is high, could enhance the number of association discoveries. This effect is amplified when the results from M-GWAS and B-GWAS2 are compared.

### 4.2. Real Study

The M-GWAS applied by chromosomes selected 1031 associated SNPs for the first seven traits (SC, CW, ADG, DP, HW, BW and PH) under study. Their distribution across the genome is displayed in Figure 2. Most of them were located in the BTAs 2, 6, 7, and 15 with more than 50 SNPs in total. The remaining markers were about evenly distributed in the other chromosomes. For each trait, the maximum number of linearly independent SNPs was around 190 (Table 3). The CDA developed by using those markers perfectly separated the LP from the HP for the seven traits, and the DA correctly assigned all the animals to the true group of origin. Moreover, the M-GWAS was able to detect 60 over 96 SNPs that were declared associated in the T-GWAS.

Considering one of the seven traits, BW for example, the 191 selected SNPs perfectly captured the differences between the animals belonging to the LP and HP. However, not all SNPs equally contribute in separating groups. Markers with greater CNC absolute values have a more important role in discriminating groups than those with lower CNCs. According to this suggestion, the minimum number of markers, for each phenotype, able to discriminate groups was obtained (Table 3) by deleting, in a recursive procedure, SNPs with lower CNC. For BW, 94 over 191 SNPs were enough to significantly discriminate LP from HP and to correctly assign all the animals to the two groups. In our opinion, the minimum number of SNPs able to significantly separate groups would be considered “significantly” associated to the trait under study.

In the T-GWAS developed by Sorbolini et al. [15], SW, CC and pH24h, did not have any associated marker. In the present study, the M-GWAS selected 141, 148 and 176 linearly independent markers for SW, CC and pH24h, able to significantly separate LP from HP. The minimum number of SNPs able to discriminate groups was quite high with respect the first seven traits, 122, 129 and 142 for SW, CC and pH24h, respectively. This could be explained by the fact that the M-GWAS has a great power compared to T-GWAS in detecting SNPs, that does not explain a large proportion of the variance in the trait (as indicated by the large number of markers needed by the DA to correctly discriminate animals of the two groups).

Finally, for the 10 traits, a pool of markers with a CNC absolute value greater than 0.25 was selected. This threshold allowed to obtain a restricted number of highly discriminant markers (108) to submit to gene discovery analysis. Their distribution is reported in Figure 3. Among the 108 SNPs, only 10 highly discriminant markers were also found with the T-GWAS. This result indicates that the multivariate approach captures the differences between HP and LP using those markers that, acting together, are able to better separate the two groups. Therefore, a single SNP can have a very little impact on the trait but, acting together with other markers, it can be very important in discriminating groups.

The number of highly discriminant SNPs obtained for SW, CC and pH24h (Figure 3) was lower than those selected for the traits that in the T-GWAS had associated markers. This result could be related to the determinism of the trait.

The gene discovery analysis conducted on the 108 most discriminant SNPs highlighted several interesting candidate genes, only in the nearby of 76 SNPs. Table 4 displays, for each chromosome, the trait and the 76 associated markers. For example, on BTA2 at 20,813,843 bp the BTB-00083120 several members of the Homeobox gene family was significant associated with SC. These genes were involved in the differentiation and development of limbs [24] and mutations in this gene have been associated with severe developmental defects on the anterior-posterior limb axis [25]. Moreover, for the ADG, CW and DP traits, some genes controlling nutrient metabolism were detected: *APLNR* and *TKT* loci are involved in the glucose metabolism, whereas SLC6A6 gene was related to the taurine transmembrane transport activity.

Among the genes highlighted for CC, *TBC1D5* was reported to be associated with the sum of omega-3 and the relative growth rate [26] and with lactation persistency [27]. For the three genes retrieved for SW, Howard et al. [28] highlighted a possible connection with heat stress: *RIPK1*, associated with cellular response to stress and apoptosis; *TUBB2B*, associated with gap junction; *SLC22A23*, associated with ion transport. Finally, the *AANAT* gene was flagged for pH24h: this gene was associated with the muscle fatty acid profile [29], collagen and pH thaw and carcass traits [30].

## 5. Conclusions

In the present research, a multivariate approach to develop a GWAS was proposed. The results of the simulation study highlighted the ability of M-GWAS in detecting the true associated markers and QTLs, especially when a small number of animals are genotyped and phenotyped. The proposed approach was more efficient than T-GWAS and B-GWAS1, also when the number of available individuals increases. Performances of M-GWAS and T-GWAS converge when more than 8000 animals are used. The B-GWAS2 approach gave results sometimes better than the M-GWAS, however, B-GWAS2 requires the number of associated SNP to be previously fixed by M-GWAS. The proposed methods detected, at least in part, QTLs in different regions of the genome. Therefore, their combined use, especially when the number of involved animals is high (more than 5000) could enhance the number of discoveries. The ability of M-GWAS in detecting associations was also confirmed when real data was used. For the first seven traits, the multivariate approach found around 60% of the associated markers obtained with the T-GWAS. Moreover, associations were detected by the M-GWAS also for the last three traits that, in the traditional approach, did not have any significant SNPs. The reliability of those associations was verified by the gene discovery analysis that was carried out on most discriminant markers.

## Figures and Tables

**Table 1:** Number of detected QTLs, false and true associated markers in the simulated dataset as the number of involved animals increases both for the multivariate (M-GWAS) and the traditional (M-GWAS) genome-wide association studies (GWAS).

Animals	M-GWAS			T-GWAS		
	Associated SNP	True Associated SNP	QTL ^1^	Associated SNP	True Associated SNP	QTL
250	110	4	4	0	0	0
500	300	18	12	0	0	0
750	450	27	20	0	0	0
1000	650	41	20	2	1	1
1250	840	47	22	11	4	2
1500	1130	55	31	38	8	4
1750	1310	72	29	120	12	4
2000	1540	92	46	130	22	10
2500	2000	94	45	240	21	7
3000	2500	127	65	359	29	13
3500	3050	157	73	1331	73	32
4000	3300	141	69	1816	96	41
4500	3700	176	82	2209	113	54
5000	4300	217	96	2713	170	73
6000	5000	225	105	3052	166	71
7000	5500	249	102	4578	226	87
8000	7000	321	114	6217	299	116
9000	7500	336	119	7313	339	121

^1^ Number of QTLs in the population = 203.

**Table 2:** Number of detected QTLs, false and true associated markers in the simulated dataset as the number of involved animals increases for both Bayesian (B-GWAS1 and B-GWAS2) GWAS methods.

Animals	B-GWAS1			B-GWAS2		
	Associated SNP	True Associated SNP	QTL ^1^	Associated SNP	True Associated SNP	QTL
250	0	0	0	110	2	2
500	1	0	0	300	15	14
750	0	0	0	450	30	20
1000	2	1	1	650	55	33
1250	4	1	1	840	38	27
1500	7	1	1	1130	49	32
1750	8	1	1	1310	54	32
2000	14	2	2	1540	99	58
2500	14	3	3	2000	107	64
3000	20	4	3	2500	119	63
3500	21	3	3	3050	161	81
4000	32	4	3	3300	190	94
4500	32	5	5	3700	185	80
5000	41	9	6	4300	263	115
6000	45	8	7	5000	286	102
7000	55	8	7	5500	305	124
8000	57	10	7	7000	383	144
9000	67	8	7	7500	406	151

^1^ Number of QTLs in the population = 203.

**Table 3:** Number of common QTLs detected by M-GWAS and T-GWAS, B-GWAS1 and B-GWAS2.

	M-GWAS vs.		
Animals	T-GWAS	B-GWAS1	B-GWAS2
250	–	–	0
500	–	–	4
750	–	–	5
1000	1	1	9
1250	1	1	7
1500	2	1	13
1750	1	1	9
2000	5	1	20
2500	4	2	20
3000	6	2	28
3500	17	2	39
4000	19	2	41
4500	26	4	34
5000	42	5	60
6000	45	5	58
7000	53	6	68
8000	67	5	82
9000	78	5	90

**Table 4:** Number of associated markers both in traditional and multivariate GWAS and in common markers (T-GWAS and M-GWAS, respectively). The minimum number of discriminant SNPs in the multivariate approach are also displayed.

Trait	T-GWAS SNP	M-GWAS SNP	M-GWAS v.s T-GWAS SNP	Minimum Number of M-GWAS SNP
BW ^1^	5	191	4	94
ADG ^2^	45	193	30	139
CW ^3^	9	191	4	98
DP ^4^	12	191	10	98
SC ^5^	13	193	6	108
HW ^6^	7	192	5	99
pH ^7^	5	190	1	120

T-GWAS = traditional GWAS; M-GWAS = multivariate GWAS, BW ^1^ = body weight, ADG ^2^ = average daily gain, CW ^3^ = carcass weight, DP ^4^ = dressing percentage, SC ^5^ = shank circumference, HW ^6^ = head weight, pH ^7^ = pH at slaughter.

## Supplementary Materials

The following are available online at https://www.mdpi.com/2076-2615/10/8/1300/s1. Table S1: List of the 76 most associated markers to the respective trait that had genes in their boundaries.
