# Supplementary material for: Use of the Multivariate Discriminant Analysis for Genome-Wide Association Studies in Cattle
Source: Animals (Basel). 2020 Jul 29;10(8):1300. doi: 10.3390/ani10081300 (PMC7460480; doi:10.3390/ani10081300)
Supplement: Supplementary file 1 [file animals-10-01300-s001.zip › animals-868017-supplementary-final/Table-S1-final.docx]

**Table S1.** List of the 76 most associated markers to the respective trait that had genes in their boundaries

| **BTA** | **Trait** | **Marker** | **Position^1^** |
| --- | --- | --- | --- |
| 1 | BW | BTA-39405-no-rs | 19,179,350 |
|  | CW | ARS-BFGL-NGS-22768 | 142,642,593 |
|  | DP | ARS-BFGL-NGS-24057 | 54,274,662 |
|  | CC | ARS-BFGL-NGS-115711 | 155,587,367 |
| 2 | DP | BTB-00077456 | 1,200,474 |
|  |  | ARS-BFGL-NGS-67309 | 118,498,848 |
|  |  | BTA-110873-no-rs | 118,617,225 |
|  | SC | ARS-BFGL-NGS-71755 | 18,057,166 |
|  |  | BTB-02054371 | 18,212,187 |
|  |  | Hapmap25114-BTA-49906 | 18,342,153 |
|  |  | BTB-00083120 | 20,813,843 |
|  |  | Hapmap47640-BTA-49632 | 18,693,590 |
|  |  | ARS-BFGL-NGS-98126 | 127,759,769 |
|  | SW | ARS-BFGL-NGS-41994 | 132,408,866 |
| 3 | SC | ARS-BFGL-NGS-119921 | 15,114,778 |
|  |  | BTA-67383-no-rs | 33,634,546 |
|  | PH | Hapmap60708-rs29011181 | 54,030,317 |
|  | HW | ARS-BFGL-NGS-65126 | 121,275,721 |
| 4 | ADG, BW | BTB-00182742 | 48,129,941 |
|  | SC | ARS-BFGL-NGS-21411 | 77,278,307 |
| 6 | ADG, CW | ARS-BFGL-NGS-642 | 35,843,840 |
|  | SC | BTA-77725-no-rs | 107,923,653 |
| 7 | HW | BTB-00309643 | 44,804,507 |
|  | SC | Hapmap41358-BTA-79117 | 52,462,171 |
|  |  | Hapmap27181-BTA-148757 | 75,355,176 |
|  | ADG | ARS-BFGL-NGS-6029 | 63,709,982 |
| 8 | PH | BTA-81053-no-rs | 41,868,874 |
|  | ADG, CC | ARS-BFGL-NGS-1517 | 96,550,119 |
| 9 | HW | BTB-00380633 | 15,641,092 |
|  | CW | Hapmap40657-BTA-115707 | 21,917,247 |
|  | pH24h | Hapmap54313-rs29012632 | 85,062,846 |
|  | SW | BTB-01247353 | 54,497,531 |
| 10 | ADG | BTB-01855834 | 2,818,174 |
|  | ADG, BW | ARS-BFGL-NGS-57821 | 6,248,185 |
|  |  | ARS-BFGL-NGS-116295 | 13,315,665 |
|  | pH24h | ARS-BFGL-NGS-48568 | 75,716,786 |
| 11 | SW | Hapmap40850-BTA-98774 | 42,942,455 |
| 12 | DP | BTA-115056-no-rs | 23,301,763 |
| 13 | HW | Hapmap44369-BTA-32763 | 44,719,822 |
|  |  | Hapmap47850-BTA-118310 | 59,133,064 |
|  | CW | ARS-BFGL-NGS-112445 | 53,489,743 |
| 14 | HW | Hapmap33635-BTC-049051 | 5,318,260 |
| 15 | DP | BTB-01279624 | 67,634,199 |
|  | PH | ARS-BFGL-NGS-119303 | 74,153,038 |
|  | ADG | BTB-00619772 | 81,547,653 |
|  | SC | ARS-BFGL-NGS-115316 | 76,926,260 |
|  |  | ARS-BFGL-NGS-28904 | 81,848,689 |
|  | CC | Hapmap48636-BTA-118794 | 63,156,963 |
|  |  | ARS-BFGL-NGS-73835 | 80,839,716 |
| 16 | SC | Hapmap48734-BTA-38315 | 23,845,036 |
|  | SC | ARS-BFGL-NGS-57549 | 43,299,381 |
|  | HW | BTB-01495723 | 38,116,695 |
|  | CC | BTB-00652797 | 61,774,249 |
| 17 | DP | ARS-BFGL-NGS-38778 | 64,452,955 |
| 18 | DP | ARS-BFGL-NGS-24323 | 36,274,916 |
|  | PH | ARS-BFGL-NGS-24006 | 15,971,297 |
| 19 | HW | ARS-BFGL-NGS-93006 | 25,477,190 |
|  |  | ARS-BFGL-NGS-117951 | 53,162,864 |
|  | SC | ARS-BFGL-NGS-112332 | 56,497,918 |
|  | pH24h | UA-IFASA-6029 | 55,900,714 |
| 20 | SC | BTB-00778405 | 37,103,943 |
|  | HW | ARS-BFGL-NGS-117598 | 73,496,969 |
| 21 | DP | ARS-BFGL-NGS-115704 | 14,510,023 |
| 22 | CW | UA-IFASA-6018 | 48,206,884 |
|  |  | BTA-54868-no-rs | 56,868,904 |
|  | CW, BW | Hapmap41774-BTA-121358 | 48,282,179 |
|  | ADG | ARS-BFGL-NGS-76123 | 49,493,685 |
|  | DP | Hapmap26413-BTA-146026 | 57,827,442 |
| 23 | PH | Hapmap38418-BTA-57213 | 9,143,924 |
|  | SW | ARS-BFGL-NGS-103489 | 50,241,214 |
| 24 | CW | Hapmap38513-BTA-58574 | 51,201,345 |
|  | DP | BTB-00856713 | 58,434,298 |
| 27 | pH24h | ARS-BFGL-NGS-15147 | 37,628,869 |
| 29 | SC | Hapmap54633-rs29021971 | 25,262,747 |
|  |  | ARS-BFGL-NGS-4431 | 38,371,072 |
|  | pH24h | BTB-01037943 | 50,512,108 |

BW=body weight, ADG=average daily gain, CW=carcass weight, DP=dressing percentage, SC=shank circumference, HW=head weight, pH=pH at slaughter, CC = carcass conformation, SW = skin weight, pH24h = pH at 24 hours after slaughter. ^1^SNP were mapped on the UMD 3.1.1 release of the bovine genome assembly
