# Supplementary material for: Use of the Multivariate Discriminant Analysis for Genome-Wide Association Studies in Cattle
Source: Animals (Basel). 2020 Jul 29;10(8):1300. doi: 10.3390/ani10081300 (PMC7460480; doi:10.3390/ani10081300)
Supplement: Supplementary file 1 [file animals-10-01300-s001.zip › animals-868017-supplementary-final/Table-S2-final.docx]

Supplementary material: SAS code.

In this example, data for a random sample of 250 animals were analyzed. Only the first two chromosomes were used.

The starting dataset was *pheno* which contained four columns: the animal i.d. (id), the generation (G), the sex (S), and the phenotype (phen)

/* the phenotype was corrected for fixed effects of generation and sex and for the random effect of animal*/

**proc** **mixed** data=pheno update info; class G S id;

model phen= G S/ DDFM=bw solution CL residual outp=predresid; random id;**run**;

/*residuals were considered the corrected phenotype (corphen)*/

**data** predresid; set predresid; rename resid=corphen; **run**;

/*a new dataset *class* was generated. It contained the two phenotype classes: low phenotype LP=1 and high phenotype HP=3 */

**data** class; set predresid; keep id phen; **run**;

**proc** **sort** data=class; by phen; n+1; **run**;

**data** class; set class; if n<=125 then cl=**1**; else cl=**3**; **run**;

/*The 2 chromosomes, *c1 and c2*, each containing the animal i.d. and the genotypes (var1, var2,……..) were then uploaded. For each chromosome, after the variable class was added, the canonical discriminant analysis was developed and, from *st* file, the canonical coefficients cnc were extracted. For each chromosome, a variable ch indicating the chromosome was added to each dataset */

%let n=1;

**proc** **sort** data= c&n; by id; **run**; (1)

**proc** **sort** data= class; by id; **run**;

**data** c&n; merge c&n class; by id; **run**;

**proc** **candisc** data=c&n distance anova out=outcan short outstat=st;

class cl; var var2--var3648 ; **run**;

**data** st; set st; if _type_ ne 'STRUCTUR' then delete; **run**;

**proc** **transpose** data=st out=st&n; var var2--var3648 ;**run**;

**data** st&n; set st&n; cnc=abs(can1) ;**run**;

**data** st&n; set st&n; drop can1; rename _name_ =snp; **run**;

**proc** **sort** data=st&n; by descending cnc; **run**;

**data** st&n; set st&n; n+**1**; **run**;

**data** st&n; set st&n; if n>100 then delete; **run**; (2)

**data** st&n; set st&n; ch=&n; **run**;

%let n=**2**;

**proc** **sort** data= c&n; by id; **run**;

**proc** **sort** data= class; by id; **run**;

**data** c&n; merge c&n class; by id; **run**;

**proc** **candisc** data=c&n distance anova out=outcan short outstat=st;

class cl; var var2--var3023 ; **run**;

**data** st; set st; if _type_ ne 'STRUCTUR' then delete; **run**;

**proc** **transpose** data=st out=st&n; var var2--var3023 ;**run**;

**data** st&n; set st&n; cnc=abs(can1) ;**run**;

**data** st&n; set st&n; drop can1; rename _name_ =snp; **run**;

**proc** **sort** data=st&n; by descending cnc; **run**;

**data** st&n; set st&n; n+**1**; **run**;

**data** st&n; set st&n; if n>**100** then delete; **run**;

**data** st&n; set st&n; ch=&n; **run**;

/* The 2 datasets were then appended */

**data** st; set st1; **run**;

**proc** **append** base=st data=st2; **run**;

/*a new variable snp was added to *st**/

**data** st; set st; m='snp'; **run**;

**proc** **sort** data=st; by ch snp; **run**;

**data** st; set st; n+**1** ; **RUN**;

**data** st; set st; mark=compress(m||n); **RUN**;

**data** st; set st; drop m n; **run**;

**proc** **sort** data=st; by ch snp; **run**;

/* the 2 chromosomes were transposed thus obtaining 2 new datasets, *cc1 and cc2.**/

%let n=1;

**proc** **transpose** data=c&n out=cc&n; var var2--var3648 phen; by id; **run**;

**data** cc&n; set cc&n; ch=&n; **run**;

%let n=2;

**proc** **transpose** data= c&n out=cc&n; var var2--var3023 phen ; by id; **run**;

**data** cc&n; set cc&n; ch=&n; **run**;

/*The 2 files were appended thus obtaining the *ok* dataset*/

**data** ok; set cc1; **run**;

**proc** **append** base= ok data= cc2; **run**;

/* columns in the dataset *ok* were correctly named and the class variable was added*/

**data** ok; set ok; rename _name_=snp; rename col1=genotype; **run**;

**proc** **sort** data=ok; by ch snp; **run**;

**data** ok; merge ok st; by ch snp; **run**;

**data** ok; set ok; if cnc=**.** then delete; **run**;

**proc** **sort** data=class; by id;**run**;

**proc** **sort** data=ok; by id; **run**;

**data** ok; merge ok class; by id; **run**;

**data** a; set ok;**run**;

**proc** **sort** data=a; by mark; **run**;

**data** b; set a; **run**;

**proc** **sort** data=b nodupkey; by mark; **run**;

**data** b; set b; order=substr(mark, **4**); **run**;

**data** b; set b; order=order***1**; **run**;

**proc** **sort** data=b; by order; **run**;

**data** b; set b; n=**1**; **run**;

**data** _null_; set b; by n; if first.n;call symput('begin', mark); **run**;

**data** _null_; set b; by n; if last.n;call symput('end', mark); **run**;

%put &begin; %put &end; %let primo=&begin; %let ultimo=&end;

**data** b; set b; keep mark order; **run**;

**proc** **sort** data=b; by mark; **run**;

**data** c; merge a b; by mark; **run**;

**proc** **sort** data=c; by id cl order ; **run**;

**proc** **transpose** data=c out=out; var genotype;id mark; by id cl; **run**;

**data** out;set out; keep id cl &primo--&ultimo; **run**;

/*the stepwise discriminant procedure was developed to obtain the maximum number of linearly independent markers. With this aim, Pr2e was fixed as 0.000000…2. With the retained markers, the canonical discriminant analysis and the discriminant analysis were performed*/

%let _stdvar = ;

**proc** **stepdisc** data=out method=Fw pr2e=**0.0000000000000002** short ; class cl;

var &primo--&ultimo; ods output summary=sum; **run**;

**proc** **candisc** data=out distance anova out=outcan short outstat=sta; class cl;

var &_stdvar; **run**;

**data** outcan; set outcan; dummy=**2**; **run**;

**proc** **gplot** data=outcan; plot dummy*can1=cl; **run**; **quit**;

**proc** **discrim** data=out ;class cl; var &_stdvar ; **run**;

/*with the following datasteps the selected markers with their canonical coefficients (cnc) were obtained*/

**data** stat; set sta; if _type_ ne 'STRUCTUR' then delete; **run**;

**proc** **transpose** data= stat out=outstat; var &_stdvar; **run**;

**data** outstat; set outstat; rename _name_=snp; **run**;

**proc** **sort** data=outstat; by snp; **run**;

**data** outstat; set outstat; cnc=abs(can1); **run**;

**data** snpok; set c; keep snp ch mark; **run**;

**proc** **sort** data=snpok nodupkey; by mark; **run**;

**data** outstat; set outstat; rename snp=mark; **run**;

**proc** **sort** data=outstat; by mark run;

**data** snpok; merge snpok outstat; by mark; **run**;

**data** snpok; set snpok; drop can1; **run**;

| 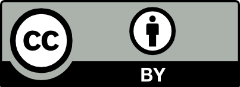 | © 2020 by the authors. Submitted for possible open access publication under the terms and conditions of the Creative Commons Attribution (CC BY) license (http://creativecommons.org/licenses/by/4.0/). |
| --- | --- |
